# Novel Ag/Si composite particles through galvanic displacement and its conductive application

**DOI:** 10.1186/s40064-016-3223-0

**Published:** 2016-09-13

**Authors:** Chenfan Yang, Xuelong Liu, Tiezheng Lv, Lili Zhao, Can Cui, Yuying Wang, Limei Cha

**Affiliations:** 1 College of Materials Science and Engineering, Hunan University, Changsha, 410012 Hunan China; 2 Department of Applied Chemistry, Harbin Institute of Technology, Harbin, 150001 China

**Keywords:** Composites, Chemical synthesis, X-ray diffraction, Transmission electron microscopy (TEM), Electrical properties

## Abstract

Here we synthesized a novel Ag/Si composite sub-micro particle using galvanic displacement by capitalizing on the active chemical surface of Si particles sludge from wafer-slicing process. Si works as chemical reactant, as well as reaction site to form composite particles. Sequent structural characterizations and analysis which include X-ray diffraction, transmission electron microscopy, scanning electron microscope, energy dispersive X-ray and electrical properties of this composite particle were done. A well-proved hetero-epitaxial growth mechanism could explain Ag nano-island/layer with a satisfactory bond property deposited on the Si surface. Since these Si are mechanically cleaved from crystal, formed conductive Ag/Si composites retain the flake shape from Si sludge particles, and narrow size distribution. They are preferred as conductive fillers, an Ag/Si composite-based conductive ink was prepared, its conductance was tested through screen printing, film thickness and resistivity were measured. The resistivity reached the µΩ cm level, even without optimizing the ink formulation. Our methods not only convert this Si sludge into highly conductive composite particles as filler for applications, but also considerably reduce the consumption of precious metal.

## Background

The synthesis, characterization of precious metal particles have been extensively studied and used in various applications. For example, Au wires are used for Integrated Circuit, light-emitting diode (LED) chip packaging and various bio-applications (Schröter et al. 2011; Chen and Goodman 2004), Ag paste is used in solar cell metallization and for electrical connection in many types modern devices (Mette et al. 2007; Roberson et al. 2015), and Pt is used as a fuel cell electrode catalyst (Stamenkovic et al. 2007). These metals have excellent conductivity, stable chemical properties, but their reserves on Earth are limited, thus are expensive. For decades, researchers have been efficiently using precious metals (Chancerel et al. 2009) or attempting to discover inexpensive replacements for these precious metals for use in existing applications (Felix et al. 2008). In the applications of catalysts for fuel cells, researchers have studied the synthesis, and electrochemical performance of hybrid Pt/Pd nanoparticles and demonstrated that these nanoparticles have superior catalytic activity and high stability (Peng et al. 2009). Ag electrodes are replaced with Cu or Ni plating on the solar cell surface (Lennon et al. 2013). For most common conductive applications in electronic devices, Ag/Cu shell/core hybrid particle-based conductive paste is used to reduce costs (Choi and Lee 2015).

Precious metal particles are normally synthesized by colloid chemical reduction method (Sau and Rogach 2010). Subsequent downstream products, such as Ag conductive paste, can be made using fine Ag particles. However, during the syntheses of precious metal-based nanoparticles, the atoms reduced from a precursor spontaneously form nuclei with different shapes and sizes, finally form particles. If no dispersant or seed is used in the nanoparticle synthesis, the shape and size of metal nanoparticles are difficult to control. Several research results demonstrated the shape-controlled synthesis of precious metal nanocrystals under thermodynamically and kinetically controlled conditions (Xia et al. 2015; Vigderman et al. 2012).

Doped Si wafers are widely used in Ultra Large Scale Integration (ULSI) and photovoltaic industries. To produce wafers, Si ingots are mechanically sliced, and polished. In the mechanical slicing process, some Si parts are crushed into particles by grits and carried away by aqueous slurry and eventually forming Si sludge. This is defined as Si kerf-loss (Moeller 2006). Compared with other Si byproducts, Si sludge not only retains the properties of the Si ingot matrix, such as satisfactory purity and resistivity, but also has several features for making novel material. Si sludge particles already have sub-micrometer dimensions, with D50 of approximately 1–2 µm (Drouiche et al. 2014). During the sawing process, Si cleavages actually occur on crystal structures and mostly become the flake shape. Most importantly, the particles retain the electrochemical properties of Si, but have a more active surface area than a bulk substrate does. Mass production of photovoltaic wafers causes considerable solid pollution because of Si sludge, however, suitable applications for this waste material are under development.

Galvanic displacement has been widely extended to the fabrication of various Si nanostructures (Peng et al. 2006; Nakamura et al. 2010; Nakamura et al. 2011), such as highly oriented Si nanowires by metal-assisted chemical etching (Huang et al. 2011). Oskam reviewed the electrochemical deposition of metals on Si substrates and demonstrated that the thermodynamics and kinetics of metal deposition on Si surfaces are dependent on several complex factors, such as the doping type of Si, concentration of HF solution, and dropping rate (Oskam et al. 1927). So far, the main research on metal–Si contacts focus on metals as contamination at the Si substrate surface during the ULSI process (Gorostiza et al. 1997). Reports on the electrochemical deposition of metals on semiconductor powder, particularly ultrafine Si powder, are scant. One promising application of Si powder in lithium ion batteries has stimulated research on the low-cost and large-scale fabrication of Si porous structures (Ge et al. 2014). In this application, the etching mechanisms are the same as etching bulk Si, but with different compositions of etchants. The deposition of precious metals with a high metal–Si ratio on Si powder has not been experimentally reported yet.

**Fig. 1:**
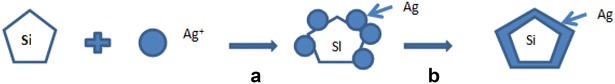
**a** Illustration of Galvanic reaction of Si particle/Ag ion and **b** the formation of Ag-Si shell/core composite structure

Here, we describe a method to fabricate highly conductive hybrid metal/semiconductor particles by using the active surface of Si sludge powder for galvanic displacement, and these Ag/Si composite particles are used for conductive applications in electronic devices. In other words, the deposition process proceeds through a galvanic displacement reaction of Ag ions that react with the surface of Si particles, then bind to form Ag nano-island, after further reactions, the Si particle is gradually covered with Ag that finally forms an Ag film, as displayed in Fig. 1. This galvanic reaction is easy to perform to a conductive external film efficiently, aqueous HF is a crucial environment for the reaction, and several properties of the resultant composite particles are determined by the Si sludge matrix.

## Experimental

### Ag/Si composite particle formation and characterizations

**Fig. 2:**
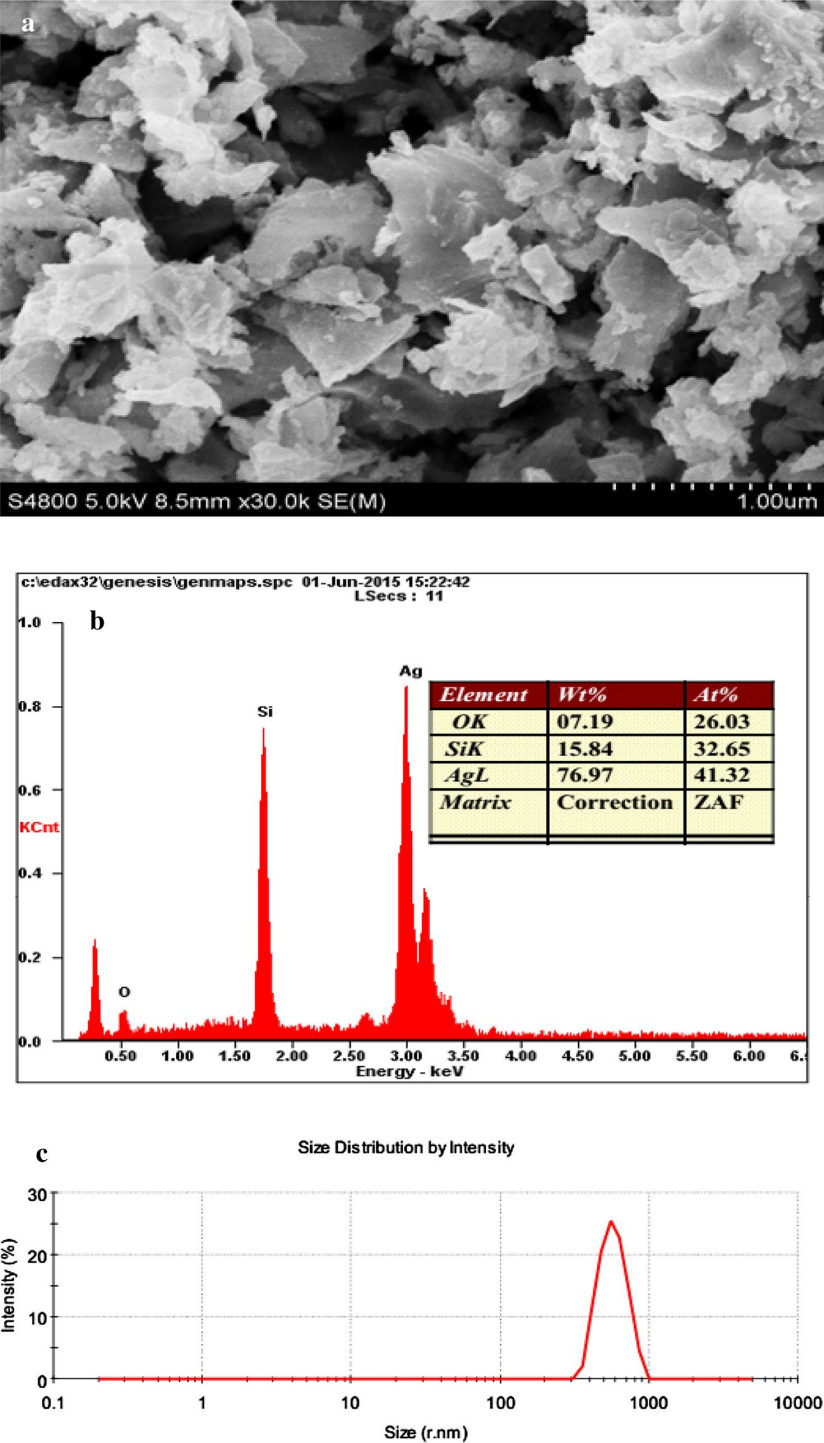
**a** SEM image of the Ag-Si composite particles, particle keeps original flake shape; **b** EDX pattern of the Ag–Si composite particle confirm the co-existed Ag and Si; **c** Size distribution of these composite particles which show the identity with initial Si sludge particles

**Fig. 3:**
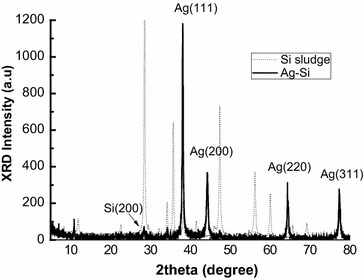
XRD patterns of finished Ag–Si composite particle (*solid line*) and the original Si sludge (*dash line*)

**Fig. 4:**
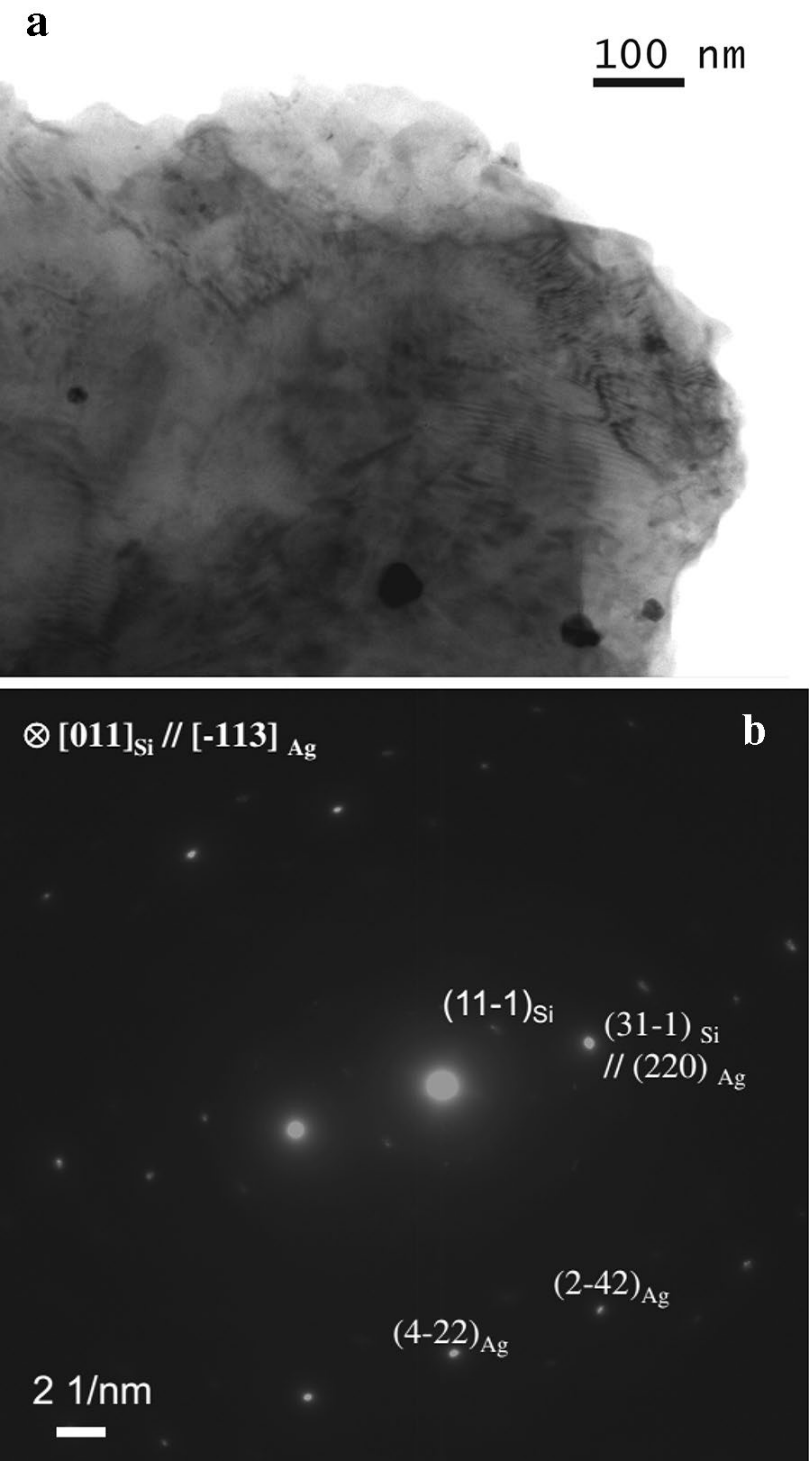
**a** BF TEM image of Ag particle/layer on Si flake. **b** SAED pattern taken from a thick flake particle, according to the index results, the crystal orientation relationship between Si and Ag is determined as [011]_Si_//[−113]_Ag_ and (31-1)_Si_//(220)_Ag_

The galvanic reaction of Ag–Si on the surface of Si is as follows:

First, Si sludge from a diamond-wire wafering process, which was provided by GCL Solar Energy Inc., was cleaned with HCl and ethane to remove metal and organic impurities and dried. For the reaction, a specific amount of Si powder was mixed with an aqueous solution of 5 M HF at room temperature with gentle stirring. However, Si powder particles are covered by silicon oxides on the surface. Oxide etching occurs rapidly when the powder is immersed in HF solution; subsequently, bubbles are generated. To suppress foam formation because of the hydrophobic character of hydrogen-terminated Si in HF solution, a small amount of ethanol is added under continuous stirring. After etching away oxides with HF solution, we dropwise added AgF solution into the aforementioned HF/Si reaction solution. Here sufficient Ag^+^ was used, normally more than the amount of participated Si. The color of as-synthesized samples changed to brownish or gray, implying that the reaction occurred on the powder surface. Scanning electron microscopy (SEM)/energy-dispersive X-ray spectroscopy (EDX) displayed the morphology of the hybrid Ag–Si structure and composition analysis provided in Fig. 2a, b. To confirm that the Ag–Si composite particles had the characteristic size of the Si matrix particles, particle size distribution analysis was performed as displayed in Fig. 2c. We were using FEI QuANTA-200 for SEM and EDX analysis, and Zetasizer NanoZS to evaluate the particle size distribution. In order to further understand the structure of this composite material, X-ray diffraction (XRD) scans on these samples were measured using D8-Advance, Bruker Inc Ag–Si crystalline structure can be confirmed from Fig. 3. TEM images in Fig. 4 were acquired using a Tecnai G2 F30 electron microscope under an accelerating voltage of 300 keV. TEM samples were prepared by a drop of a diluted dispersion of the as-formed products on copper grids, followed by air drying.

### Conductance test

**Fig. 5:**
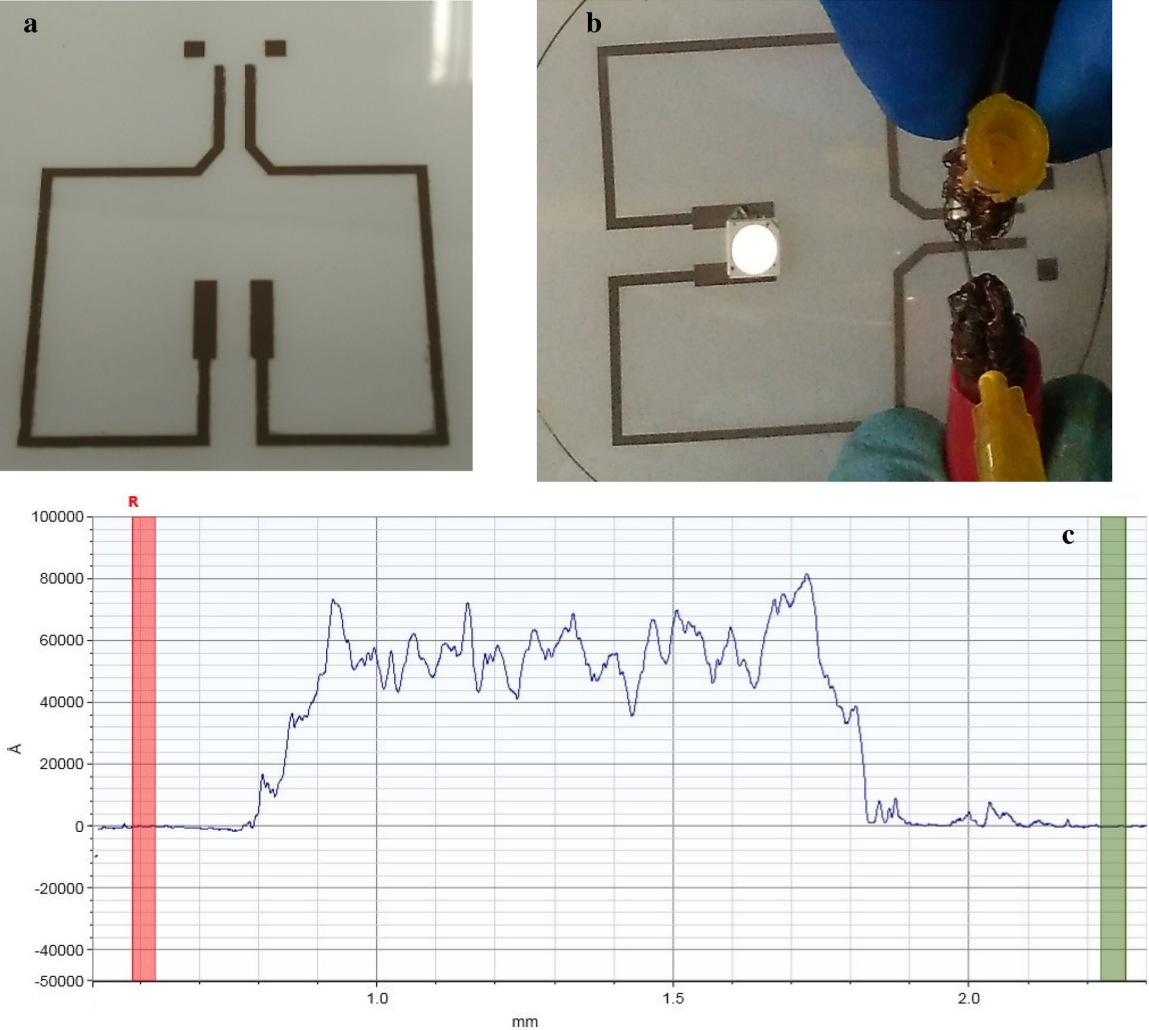
**a** Conductive line pattern made by screening printing method, **b** its conductive test by lamp lighting, **c** conductive film thickness about 5–6 µm measured by step profiler

For the application test, a water-based conductive ink with 10 % solid content was prepared following the composition in Table 1. Conductive circuits was manufactured on a plastic substrate through screen printing with a 400-mesh flatbed screen, as illustrated in Fig. 5a, and curing at a maximum of 180 °C to remove some of the organic contents for electrical tests. The electrical properties of this conductive ink were tested using a LED lamp under direct current bias, as displayed in Fig. 5b. The thickness of the printed films was 4–5 µm and measured by step profiler Dektak 150 from Veeco as illustrated in Fig. 5c. Resistivity was reached to 930 µΩ cm^−1^, using a commercial Keithley Instruments 4200 to meaure (Fig. 5).

**Table 1:** Conductive ink composition based on our particle

Composition	Percentage (%)
Hybrid solid Ag/Si particle	10
PVP dispersant	1
CMC	1
Glycerol	3
DI water	~85

## Results and discussions

Among various reducing agents for forming Ag particles, actually relying on the redox ability of them, several parameters, such as the concentration of reducing agent, temperature, and pH value as well as the dropwise sequence of solution could influence the morphological structure of the final Ag particles. Different dispersants were selected to prevent the accumulating of Ag nuclei, otherwise, Ag nuclei accumulate rapidly to form Ag particles of >10 µm and widely distribute, which is detrimental for downstream applications.

The galvanic redox process described here is straightforward and can generate Ag–Si composite particles in an easy and controllable manner. During this process, Si particles act as mild reducing agents as well as the reaction site, which the Si particle–aqueous solutions interface. Thus we could control the Ag nuclei formation on the site and prevent random nucleus formation, which occurs during the traditional redox system of producing Ag particles. The formed bundle of Ag nuclei mainly affixes to the surface of Si particles; therefore, an additional dispersant to restrict the Ag nuclei gathering process is not required.

Quasi-spherical metal nanoparticles are formed in most synthesis processes, because it is the most thermodynamically and kinetically favorable shape. To create low contact resistance among individual metal particles, we prefer synthesizing flake or wire-shaped particles, having a relatively large contact area with anisotropic growth. Here, we grew an Ag top layer on the Si core through galvanic displacement, and the flake shape of the Ag–Si composite was realized naturally and easily, almost no spherical-shaped composites were found, as displayed in Fig. 2a. This is because crystalline Si wafering process is a mechanical cleavage process occurring along the tetrahedral Si, the Si sludge forms the preferred flake shape of the final Ag–Si composite.

Figure 2b displays the approximate percentage of major elements, particularly Ag and Si in the composed particles. In conductive fillers, if approximately 15–20 % of the weight of Si can be replaced by that of Ag, as displayed in the EDX, the cost of precious metal conductive fillers can be reduced considerably. In addition to particle composition, we could confirm the particle size through PSD measurement, as demonstrated in Fig. 2c. It displays that Ag–Si composite particles have a narrow distribution, an average size D50 of approximately 800–900 nm and are completely dependent on Si matrix particles. This narrow distribution of conductive filler can prevent nozzle jam when a Ag–Si composite particle-based conductive ink is used for highly precise printed electronic devices (Mao et al. 2014).

From the XRD pattern of Fig. 3, we find this Si sludge has poly-crystalline phase, which is because of the mechanical slicing, a tough process broken Si original crystalline structure. For Ag–Si composite, the four solid peaks indicate crystalline phase of Ag, and the weak (111) peak of Si at 28.5° indicates crystallites with a deviating orientation have formed from Si core. This could prove the co-exist of crystalline Si and Ag of these samples. Figure 4a is a bright field (BF) TEM image of the composite. It shows that some dark particles with various sizes disperse on the flakes, the selected area electron diffraction (SAED) patterns in Fig. 4b were taken from a thick flake particle. According to the index results, the crystal orientation relationship between Si and Ag is determined as [011]_Si_//[−113]_Ag_ and (31-1)_Si_//(220)_Ag_. The lattice distortions are −6.13 % for Si and 6.25 % for Ag. Combing the results of TEM experiments and the XRD, which illustrates only pure Ag and Si phase exist, it could be supposed that a island or a layer/flake structure composed by Ag/Si might form additionally. Based on all the information and analysis, we can believe that crystalline Ag structures are formed on the Si matrix, and these Ag layer and/or islands structure will contribute for the conductive application.

The growth mechanism of metallic nanoparticles on semiconductor Si through galvanic displacement has a suggested mode (Sayed et al. 2009): Stranski–Krastanov mode, also called epitaxial growth mode, which is very common for lattice-mismatched hetero-epitaxial growth systems, such as Si nanowires growth using Au as a catalyst (Shimizu et al. 2007), Ag–Si interface also is investigated previously (Starr et al. 2001). In this study, we believe that hetero-epitaxial growth possibly occurred on the certain Si–Ag interface. Because of it, we assert that the Ag layer strongly bond to the Si particle matrix, and the resultant Ag–Si composite particles have excellent scratch-resistant property for applications, compared with other Ag-based shell/core particles, typically Ag/SiO_2._

For conductive applications, the measured resistivity still is higher than the ordinary resistivity of an Ag conductive ink. The difference could be attributed to low solid content or low baking temperature. This would not affect the applications if we could optimize further.

For the Ag-based conductive ink, the dispersion ability of conductive filler is a critical property because the heavy Ag particles easily sink in the conductive ink. A specific amount of light Si-composed Ag–Si composite particles reduce the actual particle density, thus the particles do not easily sink in the ink during the fabrication of electrical devices. This is another advantage for the conductive ink applications.

Two conductive theories describe the conduction mechanism of conductive filler ink applications (Vigolo et al. 2005). The contact resistance of individual particles and intrinsic resistance of a single particle have major roles in the conduction mechanism. Although the Si sludge inherits the resistivity from the mother ingots, its conductance is still much less than that of Ag; therefore, an additional thermal doping diffusion, could be performed if further improvement of the conductance of Ag–Si composite particles is needed.

Except traditional methods, we also could tune the weight ratio of the reactants Ag over Si, and the resultant Ag–Si composite particles could have an extremely broad conductance range for various applications. Finally, the Ag thin island/layer can affix well with Si flake core as Ag–Si composite particles because of a chemical reaction and more less reduce the use of noble metals for conductive applications (Hilali et al. 2006; Toriyama and Ishiwatari 2008).

According to galvanic displacement, a set of other precious metals can form the shell or islands structure on the Si sludge particle cores, indicating that a set of precious metal–Si composite materials can be produced. These materials could, in part, replace traditional precious metals in various applications. All such composite particles are worthy of investigation in other new applications, such as in surface-enhanced Raman scatterings (Cao et al. 2002) and hydrogen generation catalysts (Mckone et al. 2011).

## Conclusion

We exploited the features of ultrafine Si sludge powder as a reducing agent and a reaction site in galvanic reaction with noble metal and fabricated Ag–Si composite sub-micron structures. The structural analysis and characterizations of the fabricated particles proved that metal islands or layer could grow on the Si particle matrix. Ag–Si composites also retained the flake shape, similar to that of original Si particle matrix. A test for conductivity and measured resistivity of the composite particle-based ink revealed a satisfactory conductive property by using the Ag–Si composites as conductive fillers. Overall, the present study provides an easy, controlled process to synthesize similar metal/semiconductor composite particles that has various applications, not only for conductive ink.

Si wafer processing offers a large amount of Si sludge. Our method uses this pollutant as starting material and form valuable noble metals composite for their applications. Most importantly, these composites could substantially reduce the total use of expensive precious metals.
